# Codon-optimized *Ds*Red fluorescent protein for use in *Mycobacterium tuberculosis*

**DOI:** 10.1186/s13104-018-3798-3

**Published:** 2018-10-01

**Authors:** Paul Carroll, Julian Muwanguzi-Karugaba, Tanya Parish

**Affiliations:** 1 Queen Mary University of London, Barts & The London School of Medicine and Dentistry, London, UK; 2 Infectious Disease Research Institute, Seattle, WA 98102 USA

**Keywords:** Fluorescent protein, Mycobacteria, Reporter system

## Abstract

**Objective:**

We have previously codon-optimized a number of red fluorescent proteins for use in *Mycobacterium tuberculosis* (mCherry, tdTomato, Turbo-635). We aimed to expand this repertoire to include *Ds*Red, another widely used and flexible red fluorescent protein.

**Results:**

We generated expression constructs with a full length *DsRed* under the control of one of three strong, constitutive promoters (P_hsp60_, P_rpsA_ or P_G13_) for use in mycobacteria. We confirmed that full length *Ds*Red (225 amino acids) was expressed and fluoresced brightly. In contrast to mCherry, truncated versions of *Ds*Red lacking several amino acids at the N-terminus were not functional. Thus, we have expanded the repertoire of optimized fluorescent proteins for mycobacteria.

## Introduction

Fluorescent proteins (FPs) have become the work horses of molecular biology and microbiology, with numerous applications. A plethora of variants of *Aequorea victoria* green fluorescent protein (GFP) [1] and *Discosoma* sp red fluorescent protein (*Ds*Red) [2] are available covering almost the whole light spectrum from green to infra-red [3]. Mutant derivatives have been engineered with altered excitation and emission wavelengths, increased or decreased stability, resistance to photo bleaching, sensitivity to environmental stimuli and substrates, as well as time for fluorophore maturation, intrinsic brightness and multimeric formats [3, 4]. We previously described the use of a range of red reporters, of which the brightest was mCherry [5]. We wanted to expand our repertoire of proteins. Since *Ds*Red has been widely used as a bright and stable reporter, we optimized constructs for its expression in *M. tuberculosis*.

## Main text

### Materials and methods

#### Bacterial culture

*Escherichia coli* DH5α was cultured in LB medium or on LA agar. *M. tuberculosis* H37Rv was grown in Middlebrook 7H9 medium plus 10% v/v OADC (oleic acid, albumen, dextrose, catalase) supplement (Becton–Dickinson) and 0.05% w/v Tween 80 or on Middlebrook 7H10 agar (Becton–Dickinson) plus 10% v/v OADC. Hygromycin was used at 100 μg/ml where required.

#### Construction of expression vectors

The *Ds*Red expression vectors were constructed as follows: a partial *Ds*Red sequence was codon optimized for *M. tuberculosis*, synthesised and cloned into pUC57 (Genscript USA Inc.) to generate pRed1. The *Ds*Red ORF was excised from pUC57 as a BamHI/HindIII fragment and cloned into pSMT3 [6] to generate pBlaze1. The *Ds*Red ORF was extended three times by PCR to generate pRedA1, pRedB1 and pRedC1 using primers DsRed-F1 5′-GGA TCC **ATG** CGC TTC AAG GTG CGC ATG GAG GGC TCG GTG AAC-3′, DsRed-F2 5′-GGA TCC GAC **GTG** ATC AAG GAG TTC ATG CGC TTC AAG GTG CGC-3′ and DsRed-F3 5′-GGA TCC **ATG** GCC TCG TCG GAG GAC GTG ATC AAG GAG TTC together with the reverse primer DsRed-R 5′-AAG CTT TTA CAG GAA CAG GTG GTG CCG-3′. The restriction sites are underlined, potential start codons are in bold. The ORFs were excised and cloned into pSMT3 [6] as *Bam*HI/*Hin*dIII fragments to generate pBlazeA1, pBlazeB1 and pBlazeC1 with *Ds*Red under the control of the *hsp60* promoter (Table 1). Plasmids pBlazeC8 and pBlazeC10 were generated by replacing P_hsp60_ with P_rpsA_ and P_G13_ respectively. All three promoters should drive constitutive high level expression [5, 7, 8].

**Table 1:** Plasmids used in this study

Plasmid	Description	Promoter	Fluorescent protein	Source
pSMT3	Shuttle vector, P_hsp60_, HygR			[6]
pRED1	Codon-optimized *Ds*Red in pUC57	None	*Ds*Red208	This study
pBlaze1	*Ds*Red expression vector. HygR	Hsp60	*Ds*Red208	This study
pBlazeA1	*Ds*Red expression vector. HygR	Hsp60	*Ds*Red214	This study
pBlazeB1	*Ds*Red expression vector. HygR	Hsp60	*Ds*Red220	This study
pBlazeC1	*Ds*Red expression vector. HygR	Hsp60	*Ds*Red225	This study
pBlazeC8	*Ds*Red expression vector. HygR	RpsA	*Ds*Red225	This study
pBlazeC10	*Ds*Red expression vector. HygR	G13	*Ds*Red225	This study

#### Quantitation of fluorescence in whole cells

*Mycobacterium tuberculosis* was electroporated as described [9] and transformants selected with hygromycin. *M. tuberculosis* was grown to stationary phase, harvested, washed twice in 10 mM Tris pH 8.0 and resuspended in 10 mM Tris pH 8.0 to an OD_580_ of 0.25, 0.10, 0.05 and 0.01 in 12 × 100 mm glass culture tubes. Fluorescence was measured on a Shimadzu RF-1501 spectrofluorimeter (Shimadzu) with a detection range of 0–1015 relative fluorescent units at Ex/Em 558/583 nm [5].

#### Western analysis of fluorescent proteins

Cell extracts were prepared from liquid cultures. Cells were harvested by centrifugation, washed twice in 10 mM Tris (pH 8.0), resuspended in 1 ml of 10 mM Tris (pH 8.0), and added to lysing matrix B tubes (QBiogene). Cells were disrupted using the Fastprep (QBiogene) set at speed 6.0 for 30 s. Samples were centrifuged at 4000 rpm for two min, and the supernatant was recovered and filter sterilized (0.2 micron filter). Protein was quantified using a BCA kit (Pierce), and 10 μg of total protein was subjected to Western blot using a rabbit anti-body (Clonetech). The primary antibody was detected using horseradish peroxidase goat-anti-rabbit (Sigma), and activity was detected using an ECL kit (GE Healthcare).

### Results

We were interested in the use of FPs in *M. tuberculosis* and had previously used these as reporters of bacterial viability for in vitro and in vivo studies [5, 8]. We were successful in obtaining high level expression by using codon-optimized versions of red fluorescent proteins driven by strong mycobacterial promoters [5].

#### Optimization of *Ds*Red expression

We wanted to expand the range of reporters available for use to increase flexibility and allow dual reporter expression and monitoring. We selected *Ds*Red for optimization, based on its Ex/Em wavelengths, and the fact that it is a well-characterized FP in wide use [3, 4, 10–14].

#### Expression of *Ds*Red uses a different translational start site than mCherry

**Fig. 1:**
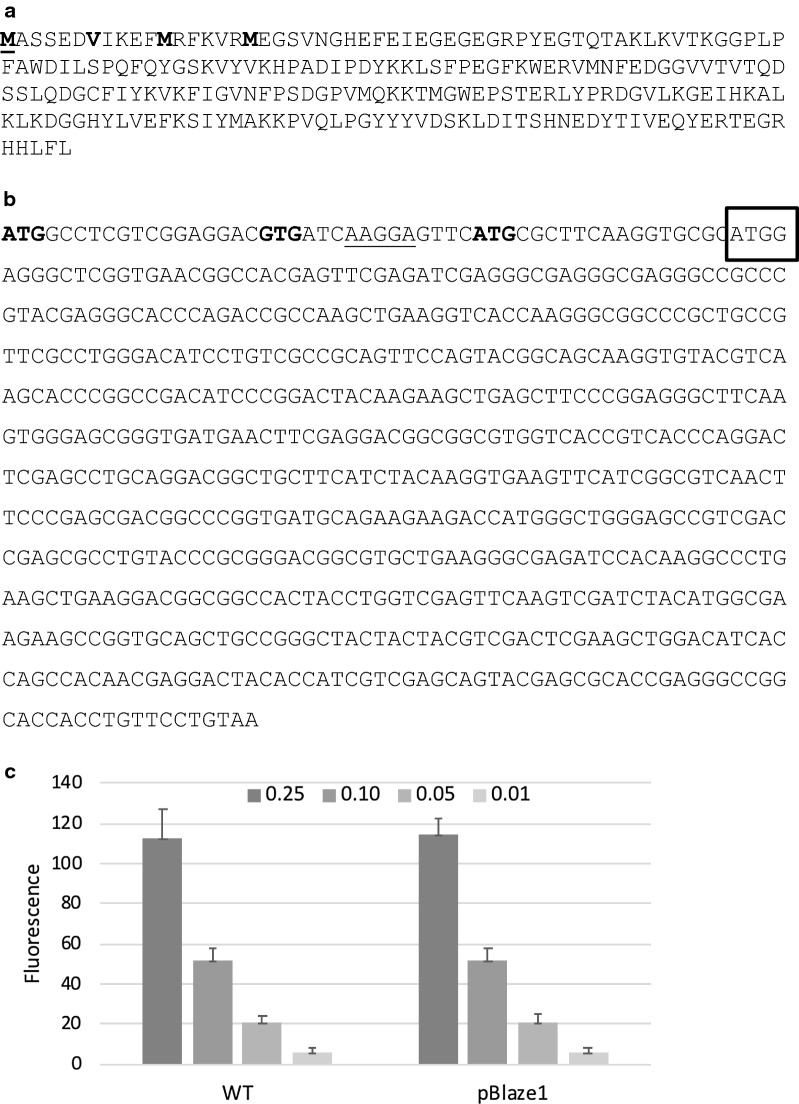
Expression of non-functional *Ds*Red. **a** *Ds*Red Protein sequence. Three potential translational start sites (methioinine) are indicated in bold. The valine which corresponds to the methionine start site of mCherry in *M. tuberculosis* is also indicated in bold. **b** DNA sequence of DsRed. The 5′ end of the synthetic gene designed to codon-optimize *Ds*Red for *M. tuberculosis* is boxed. Potential starts sites are indicated in bold. The Shine Delgarno sequence is underlined. **c** *M. tuberculosis* was resuspended in 10 mM Tris pH 8.0 to an OD_580_ of 0.25, 0.10, 0.05 and 0.01 in 12 × 100 mm glass culture tubes. Fluorescence was measured at Ex/Em 558/583 nm. WT—wild-type (no plasmid). pBlaze1—recombinant strain carrying *Ds*Red 208aa. Data are the average ± SD of three cultures

Our initial attempts to obtain expression of a codon-optimized *Ds*Red were unsuccessful. We constructed a synthetic gene for *Ds*Red using a similar approach as we used with another red fluorescent protein mCherry (Fig. 1). We designed a codon-optimized version based on the *Ds*Red-T3 protein previously used. We cloned the synthetic version into a mycobacterial expression vector and tested for fluorescence in *M. tuberculosis*. Surprisingly, we did not detect any fluorescence from this construct (Fig. 1c).

**Fig. 2:**
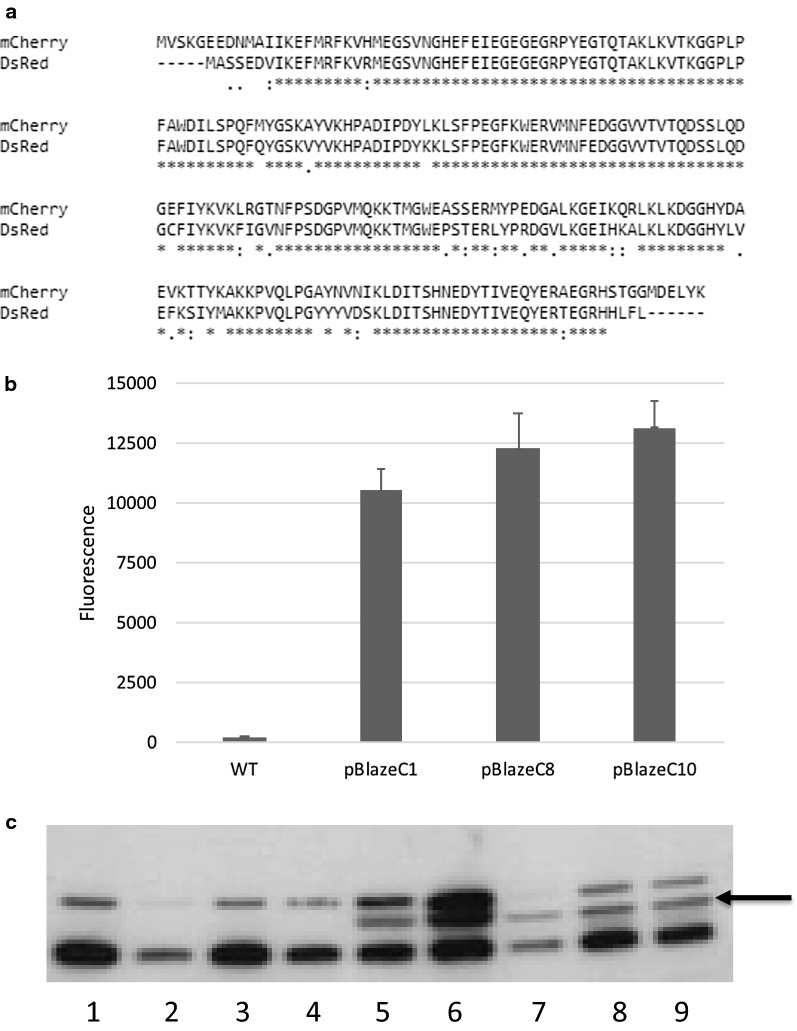
Expression of functional *Ds*Red. **a** Sequence alignment of mCherry and *Ds*Red. Protein sequences were aligned using Clustal 2.1 [19]. **b** Activity of full length *Ds*Red expressed from mycobacterial promoters. pBlazeC1-P_hsp60_; pBlazeC8-P_rpsA_; pBlazeC10-P_G13_. Recombinant *M. tuberculosis* was resuspended in 10 mM Tris pH 8.0 to an OD_580_ of 0.25, 0.10, 0.05 and 0.01 in 12 × 100 mm glass culture tubes. Fluorescence was measured at Ex/Em 558/583 nm. Data are the average ± SD of three cultures. **c** Plasmids were transformed into *E. coli* and cell-free extracts analyzed by Western blotting; 10 µg protein were subjected to SDS-PAGE, blotted onto PVDF membrane and visualized with anti-DsRed antibody. Lane 1—*E. coli* (no plasmid); Lane 2—pRed1; Lane 3—pRedA1; Lane 4—pRedB1; Lane 5—pRedC1; Lanes 6 and 7—pBlazeC1; Lane 8—pBlazeC8; Lane 9—pBlazeC10. The arrow indicates the size of the *Ds*Red protein

mCherry is a variant of *Ds*Red and we expected the two proteins would be similarly functional. Our previous work demonstrated that mCherry is expressed from a distal translational start site than the one annotated in the databases [15]. Sequence alignment shows the few mutations which differ between the two (Fig. 2a); these include loss of the translational start site we identified for mCherry, although there are still multiple translational start sites (Fig. 1a). The version we used for the synthetic gene used a downstream translation start site and would produce a truncated version of *Ds*Red as compared to mCherry. Therefore it was possible that we did not express the full protein (Fig. 1b). In order to determine the functional start site for *Ds*Red we used a different approach in which we cloned several versions of the coding region into the expression vector under the control of the constitutive *hsp60* promoter (Fig. 2b).

In order to test this, we used PCR amplification to extend the region sequentially. We extended the gene to incorporate both additional start sites and generate proteins of 214, 220 and 225 amino acids. These variants were cloned into the same mycobacterial expression system and tested. Plasmids were transformed into *M. tuberculosis* and fluorescence was monitored. In contrast to mCherry, expression of a functional fluorescent *Ds*Red was not seen with any truncated versions of the protein. In fact fluorescence could only be detected when the full length amino acid sequence (as annotated) was cloned into the expression vector; high level fluorescence was seen with transformants carrying the plasmid pBlazeC1 (Fig. 2b).

We constructed two alternative vectors with *Ds*Red under the control of either P_rpsA_ or P_G13_ (pBlazeC8 and pBlazeC10 respectively); both of these constructs gave high level expression in *M. tuberculosis.* Western blotting using an anti-*Ds*Red antibody in *E. coli* demonstrated that a protein of the expected size was only seen in bacteria carrying the full length construct (pBlazeC series), but not in the strains carrying the truncated version (Fig. 2c; lanes 5–9). Two additional bands are see in the Western, these are unknown proteins, but are also present in the control *E. coli* (no plasmid, Fig. 2c, lane 1).

### Discussion

We have determined that the functional translational start sites for two closely related FPs are different in *M. tuberculosis*. Although mCherry was functional even when a truncated version was being expressed, *Ds*Red was non-functional in a truncated form and only fluoresced when expressed as a full length protein (225 amino acids). Western blotting suggested that the lack of fluorescence was most likely due to a lack of protein expression, since proteins could not be detected in the plasmids carrying truncated forms. This difference may relate to protein stability, with the extended N-terminal portion of *Ds*Red increasing stability or protein maturation; alternatively this could be attributed to the physical state of the active proteins, since mCherry functions as a monomer, whereas *Ds*Red is a tetramer which might also affect protein degradation.

Fluorescent proteins have proved useful in multiple applications in mycobacteria; our previous constructs using mCherry have been widely disseminated to the community and used in a range of methods. For example, we have used these for high throughput drug testing [16], and imaging infection using animal models [8]. Other approaches have used mCherry to develop reporter strains for environmental sensing [17].

In conclusion, we have codon-optimized *Ds*Red for use in *M. tuberculosis* and demonstrated its high level fluorescence in that species from three different promoters of slightly varying strength (*hsp60*, *rpsA*, and *G13*). These vectors extend our current repertoire of functional fluorescent proteins for mycobacteria. They will be useful for generating fluorescent strains of *M. tuberculosis* for use in multiple studies, such as monitoring drug efficacy in vitro and in vivo [5, 8, 16, 18] and will allow for detection of multiple reporters simultaneously.

## Limitations

- We have monitored the expression of *Ds*Red under aerobic conditions only.
- We have not monitored long term stability of expression in the absence of antibiotic selection to maintain the plasmid.
- We have not monitored stability of expression in vivo.

## Abbreviations

FP: fluorescent protein
OADC: oleic acid, albumin, d-glucose, catalase
